# Supplementary material for: Strategies of property developers in the context of carbon tax
Source: PLoS One. 2023 May 4;18(5):e0283527. doi: 10.1371/journal.pone.0283527 (PMC10159139; doi:10.1371/journal.pone.0283527)
Supplement: Attachment — Submitted filename: Comments from reviewers.docx [file pone.0283527.s003.docx]

Comment a:

The author draws four conclusions in the case of activating a carbon tax, but the first two conclusions seem to have little intention. Even without corresponding model deduction, it is easy to get relevant opinions.

Comment b:

The foreshadowing in the introduction part is too wordy and does not lead to the research questions of this paper concisely and clearly, so it is not readable. In addition, the "To successfully boost property developers' emission reduction potential" used by the author has problems. The potential should be a relatively objective existence and should be to improve the efficiency of emission reduction.

Comment c:

Where the model is set up, the author asserts two types of competing property developers in the same market where products are substitutable, and refer to them as duopoly competitive property developers This duopoly competition hypothesis deviates greatly from the actual characteristics of China's real estate market. China's real estate market has experienced explosive development, and a large number of real estate enterprises have emerged. Even in the county level cities, the competitors in the real estate market are also very diversified, rather than duopoly competition. In the real estate market of cities above county level, it is a multi game between a large number of high-end brands. If the author focuses on the real estate market below the county level (towns and townships), more houses in relevant markets are built by residents themselves, which has little relevance to the research topic and has no practical significance. At the same time, the Chinese government has actively carried out anti-monopoly activities and has also issued anti-monopoly laws. The author's duopoly competition hypothesis will not appear in the future Chinese real estate market, and the relevant research results have limited significance. In addition, the author studies the real estate duopoly competition game in the context of carbon reduction. However, it is difficult to find a country whose real estate market conforms to the characteristics of duopoly competition among the major carbon emitting countries.

Comment d:

The author advocates the duopoly game model of the real estate market constructed by themselves. However, the relevant model settings do not show the particularity of the real estate market. Just changing the product name to the square number of real estate does not reflect the particularity of the real estate market. We should further consider the impact of high circulation demand, capital chain demand, government price limits, purchase restrictions and other factors of real estate developers.

Comment e:

The real estate market in different regions of China is very different, and the simulation part can be used for regional comparative analysis.
