# Supplementary material for: Strategies of property developers in the context of carbon tax
Source: PLoS One. 2023 May 4;18(5):e0283527. doi: 10.1371/journal.pone.0283527 (PMC10159139; doi:10.1371/journal.pone.0283527)
Supplement: Attachment — Submitted filename: Referee report.docx [file pone.0283527.s004.docx]

**Review report on PLOS ONE**

**" Strategies of duopoly property developers in the context of carbon tax "**

This study analyses the impact of environmental taxes on firms' pollution reduction behaviour by utilising the Stackelberg model. After deriving the equilibrium analytically, a simulation analysis with the Chinese economy in mind reveals the impact of environmental taxes on pollution emissions and firm profits. As China has not officially introduced an environmental tax, the results of this study may provide useful insights for policy makers. However, while the equilibrium of the economic model is carefully demonstrated, its econometric intuition is not well explained. Instead, the intuition is explained by focusing on the results of a simulation analysis assuming specific parameter conditions. The explanation of the intuition for the analytical results further enhances the generality of this analysis. The results further increase the persuasiveness of the simulation analysis. The authors should make an effort to increase the generality of their analysis for the acceptance of this study in this journal.

**・Major revision**

The significance of this study is not very clear. A paragraph should be added in the introduction that clearly explains what the key research questions are that previous research analyses have missed and how this study seeks to meet those questions. In doing so, the significance of the study will be clearer if it is clarified how the setting differs from existing theoretical models, and how these differences affect the results.

Section 3.2, where the comparative statics analysis is performed, is the key section comprising the main results of this study. In doing so, the authors only describe their results in each Inferences, with scant explanation of their economic intuitions. In the absence of this explanation, it is difficult to ascertain what mechanisms are at work behind the simulation analysis in Chapter 4. The authors should follow their proof with a careful explanation of their intuition about Inferences.

In Figures 3 and 4, there is a non-monotonic relationship between the profit of the property developer and the environmental tax. This relationship arises because the environmental tax creates a profit-raising effect in addition to a profit-decreasing effect. It should be intuitively explained that through these effects a non-monotonic relationship is created.

The non-monotonic relationship between profit and environmental taxes generates a threshold environmental tax. It should be proved, if possible, by what parameter changes this threshold increases or decreases. Using the results as an Inference, intuitively explain when environmental taxes become more profitable. The results would provide useful policy recommendations.

In Inference 4, the authors should explain why the effect of environmental taxes to increase profits disappears when firms have an advantage with regard to emission reduction costs.

In Inference 5, the authors should explain what parameter changes would reduce or increase this difference.

Based on the results of Inference 1-5 and the parameter changes, the authors should clarify under what economic conditions environmental taxes have a profit-increasing effect.

The additional explanations provided by this revision could be summarised as policy recommendations in the conclusion.

**Minor revision**

In the proof of Theorem 2.1.1, there is a formula with a small font size of 0 and a formula containing 且.
